# SLPI is associated with AR-regulated epithelial adaptation and enzalutamide sensitivity in castration-resistant prostate cancer

**DOI:** 10.3389/fphar.2026.1876355

**Published:** 2026-08-18

**Authors:** Zhen Zhu, Yuyong Shen, Xingjun He, Ming Zhou

**Affiliations:** Department of Urology, The Affiliated Hospital of Yangzhou University, Yangzhou University, Yangzhou, China

**Keywords:** castration-resistant prostate cancer, enzalutamide, Immunoregulation, prostate cancer, single-cell RNA sequencing, SLPI, therapeutic sensitivity, tumor ecosystem

## Abstract

**Background:**

Castration-resistant prostate cancer (CRPC) develops through tumor-cell plasticity, androgen receptor (AR) pathway rewiring, and immune/stromal changes under therapeutic pressure. Because metastatic CRPC often responds incompletely to immunotherapy and AR-directed treatment, cell-resolved analyses may help place putative drug-response factors within their tissue context. We examined whether secretory leukocyte protease inhibitor (SLPI), an epithelial secreted factor with immunoregulatory functions, is related to epithelial-state changes and enzalutamide response.

**Methods:**

Public single-cell RNA-sequencing data from localized prostate cancer and metastatic CRPC (GSE274229) were analyzed using Seurat-based preprocessing, cell-type annotation, UCell pathway/state scoring, CellChat ligand-receptor inference, CopyKAT malignant-cell inference, Monocle2 trajectory analysis, hdWGCNA network analysis, and TCGA-PRAD clinical association analysis. SLPI was evaluated in 22Rv1 and C4-2 prostate cancer models using stable overexpression, qRT-PCR, proliferation, colony formation, migration, AR-pathway modulation, enzalutamide dose-response, and Annexin V/PI apoptosis assays.

**Results:**

Progression to mCRPC was accompanied by altered epithelial-immune/stromal communication and shifts in inflammatory, extracellular-matrix, growth-factor, and immune-regulatory signaling. Putative malignant epithelial populations showed reduced AR-dependency and luminal-identity scores together with increased stress-adaptation and plasticity programs. Network and clinical association analyses highlighted SLPI as a progression-related epithelial factor with outcome relevance. *In vitro*, SLPI expression was lower in prostate cancer cells than in RWPE-1 cells, and SLPI overexpression suppressed proliferation, migration, and clonogenic growth. AR-pathway modulation showed reciprocal changes in SLPI expression: androgen deprivation and AR knockdown increased SLPI expression, whereas DHT-mediated AR reactivation reduced SLPI expression. SLPI overexpression enhanced enzalutamide-induced growth inhibition and apoptosis in the tested cell-line models.

**Conclusion:**

These findings place SLPI at the intersection of AR-pathway state, epithelial adaptation, immune-context changes, and enzalutamide sensitivity in CRPC. SLPI may represent a therapy-response-associated epithelial factor in prostate cancer, but further protein-level, loss-of-function, *in vivo*, and clinically annotated validation will be required.

## 1 Introduction

Prostate cancer remains one of the most common malignancies in men worldwide, and durable disease control is still limited by progression after systemic therapy (Bray et al., 2024; Raychaudhuri et al., 2025). Androgen deprivation therapy and next-generation AR signaling inhibitors such as enzalutamide have transformed the management of advanced prostate cancer, but resistance commonly develops after an initial response (Scher et al., 2012; Beer et al., 2014; Miller et al., 2024). This transition reflects adaptive pressure across malignant epithelial cells, immune populations, stromal niches, and soluble signaling networks that together shape pharmacologic sensitivity and resistance (Kulasegaran and Oliveira, 2024; Watson et al., 2015; Vellky and Ricke, 2020; Chaudhary et al., 2025).

Mechanistically, CRPC progression is characterized by persistent or rewired AR signaling, lineage plasticity, neuroendocrine-like differentiation, and stress-adaptive epithelial states (Beltran et al., 2016; Imamura et al., 2023). Single-cell studies have shown that prostate cancer epithelial cells can move away from canonical AR-dependent luminal programs toward stress-adapted, inflammatory, or lineage-plastic states (Taavitsainen et al., 2021; Chan et al., 2022; Song et al., 2022; Zhao et al., 2024). Recent cell-resolved prostate cancer datasets have also linked epithelial-state changes to immune and myeloid resistance programs (Lyu et al., 2025). Therefore, mechanisms of drug sensitivity in prostate cancer must be interpreted across cell types and cannot be reduced to bulk tumor-cell averages (Jiang L. et al., 2024).

SLPI is a secreted serine protease inhibitor with roles in epithelial barrier protection, protease control, antimicrobial defense, and inflammatory regulation (Nugteren and Samsom, 2021; Zhang et al., 2023; Akdere et al., 2025). These functions make SLPI relevant to treatment-response biology: as a secreted epithelial regulator, it may influence cell-intrinsic stress responses while also reflecting inflammatory and microenvironmental states. In prostate cancer, SLPI has been associated with progression, recurrence, and AR-related biology, but its relationship to epithelial remodeling and response to AR-directed therapy remains unresolved (Miyazaki et al., 2023; Yang et al., 2020).

Here, we combined single-cell analysis with functional pharmacology to identify regulators associated with CRPC adaptation. Using GSE274229, a prostate cancer single-cell dataset generated in the context of disease progression and immunotherapy resistance, we mapped epithelial, immune, myeloid, and stromal changes from localized disease to mCRPC. We then focused on genes related to malignant epithelial states, AR dependency, stress adaptation, and clinical outcome. SLPI stood out as an epithelial and immunoregulatory factor, and we tested its effects on malignant phenotypes, AR-pathway state, and enzalutamide sensitivity in prostate cancer models.

## 2 Methods

### 2.1 Data sources and preprocessing

Single-cell RNA sequencing data were obtained from the Gene Expression Omnibus (GEO; accession GSE274229), a prostate cancer dataset generated from tumors across disease states including localized prostate cancer and metastatic castration-resistant prostate cancer (mCRPC). The present analysis focused on localized prostate cancer and mCRPC samples to examine progression-associated changes under long-term therapeutic pressure. Data preprocessing was performed using Seurat, and dataset integration followed established single-cell integration principles (Hao et al., 2021; Stuart et al., 2019). Cells with low gene counts, excessive mitochondrial or hemoglobin gene percentages, or potential doublet features were excluded; cells expressing fewer than 200 genes were removed before downstream analysis. After log-normalization and scaling, highly variable genes were identified and datasets were integrated in Seurat before principal component analysis (PCA), Uniform Manifold Approximation and Projection (UMAP), and graph-based clustering. Cells passing all quality-control thresholds were retained for downstream analysis.

### 2.2 Cell type annotation

Cell types were annotated based on canonical marker genes, cluster-specific expression patterns, and established prostate tumor microenvironment cell identities (Cheng et al., 2023). Major cell populations identified included epithelial cells, T cells, B cells, myeloid cells, endothelial cells, fibroblasts, smooth muscle cells, and mast cells. Further subclustering analysis was performed for epithelial cells, T cells, and myeloid cells to resolve functional heterogeneity (He G. et al., 2024). Marker genes such as EPCAM, KRT8, and KRT18 were used to identify epithelial cells; CD3D and CD3E for T cells; MS4A1 and CD79A for B cells; LYZ and CD68 for myeloid cells; PECAM1 and VWF for endothelial cells; ACTA2 and TAGLN for smooth muscle cells; COL1A1 for fibroblasts; and TPSAB1/TPSB2-like mast-cell markers where available.

### 2.3 Cell composition and functional pathway analysis

Cell-type proportions were calculated at the sample level and compared between localized prostate cancer and mCRPC groups to characterize changes in cellular composition during disease progression. Functional pathway activity was evaluated using Hallmark gene sets from the Molecular Signatures Database (MSigDB) and the gene-set enrichment framework for curated pathway-level interpretation (Subramanian et al., 2005; Liberzon et al., 2015). Gene-set activity was quantified using UCell, which enables robust rank-based scoring of pathway programs in single-cell data (Andreatta and Carmona, 2021). These scores were compared across disease groups and major cellular compartments.

### 2.4 Cell-cell communication analysis

Cell-cell communication analysis was performed to evaluate inferred changes in intercellular signaling during prostate cancer progression. Ligand-receptor interaction networks were inferred using CellChat (Jin et al., 2021). Interaction number and interaction strength were compared between localized prostate cancer and mCRPC. Information-flow and network-centrality analyses were used to identify signaling pathways with altered activity across epithelial, immune, and stromal compartments (He R. et al., 2024). Because CellChat is an inference framework based on transcriptomic ligand-receptor expression, these results were interpreted as candidate communication programs rather than direct experimental evidence of SLPI-mediated intercellular signaling.

### 2.5 Epithelial cell subtype analysis

Given that epithelial cells represent the principal malignant compartment of prostate cancer, downstream analyses focused on epithelial populations. Epithelial cells were reclustered to identify transcriptionally distinct subtypes, including Basal_Epi, Luminal_Epi_1, Luminal_Epi_2, Luminal_Epi_3, Secretory_luminal_Epi_1/2, and stress-associated epithelial states. Subtype annotation was based on relative expression of basal markers, luminal/AR-lineage markers, secretory epithelial markers, and immediate-early/stress-response genes, together with cluster-specific differential expression patterns. To distinguish putative malignant from likely non-malignant epithelial cells, copy number variation (CNV) inference was performed using the CopyKAT algorithm (Gao et al., 2021). Cells predicted as aneuploid were considered putative malignant cells, whereas diploid cells were considered likely non-malignant. Differentiation potential was estimated using CytoTRACE to support interpretation of epithelial state heterogeneity (Gulati et al., 2020).

### 2.6 Cellular state scoring

To characterize malignant epithelial functional states, gene signature scores were calculated for AR dependency, luminal identity, stress adaptation, plasticity/EMT, and neuroendocrine-like features using UCell. The AR-dependency score was based on a published AR-activity-low/enzalutamide-resistance program (Alumkal et al., 2020). Luminal-identity, plasticity/EMT, and neuroendocrine-like scores were compiled from curated prostate epithelial lineage and treatment-resistance signatures. The stress-adaptation score was generated from stress-response, inflammatory-response, hypoxia, unfolded-protein-response, and apoptosis-related gene programs, with rank-based UCell scoring used to reduce sensitivity to sequencing depth. These scores were used to quantify epithelial pharmacologic adaptation and to relate transcriptional states to CRPC progression.

### 2.7 Trajectory analysis

To investigate potential ordering relationships between epithelial cell states, pseudotime trajectory analysis was performed using Monocle2 (Qiu et al., 2017). Cells were ordered along trajectories based on highly variable genes. Pseudotime distributions were compared across epithelial subtypes to identify potential state relationships from AR-dependent luminal states toward stress-adapted or AR-indifferent phenotypes. State score dynamics across pseudotime were also evaluated to characterize functional changes associated with tumor progression. These analyses were interpreted as computationally inferred trajectories and not as direct lineage-tracing evidence.

### 2.8 Gene co-expression network analysis

High-dimensional weighted gene co-expression network analysis (hdWGCNA) was performed on epithelial cells to identify gene programs associated with epithelial state remodeling (Morabito et al., 2023; Liu et al., 2024). Gene modules were constructed according to expression similarity, and module eigengenes were correlated with disease state and functional scores, including AR dependency, stress adaptation, and EMT/plasticity. Modules associated with progression or epithelial state traits were selected for hub-gene prioritization.

### 2.9 Differential expression analysis and candidate gene identification

Differential expression analysis between localized and mCRPC epithelial cells was performed using Seurat FindMarkers with multiple-testing correction. Epithelial differentially expressed genes were intersected with hub genes from phenotype-associated hdWGCNA modules to prioritize genes most consistently related to disease-associated epithelial remodeling. SLPI was carried forward for experimental validation because it was a secreted epithelial/immunoregulatory factor, appeared in this intersected gene set, was related to epithelial state programs, and showed the clearest nominal association with outcome among the tested genes in TCGA-PRAD.

### 2.10 Survival analysis using TCGA data

Clinical relevance was evaluated using bulk RNA-sequencing and clinical data from The Cancer Genome Atlas prostate adenocarcinoma cohort (TCGA-PRAD) (Cancer Genome Atlas Research Network, 2015). Candidate genes were analyzed by LASSO Cox regression using the glmnet R package, with ten-fold cross-validation to select the regularization parameter (Friedman et al., 2010). Hazard ratios and confidence intervals were estimated to assess associations between candidate-gene expression and survival outcomes (Bi et al., 2025).

### 2.11 Cell lines and cell culture

The human prostate cancer cell lines 22Rv1 (RRID: CVCL_1045) and LNCaP C4-2 (RRID: CVCL_4782), together with the non-tumorigenic prostate epithelial cell line RWPE-1 (RRID: CVCL_3791), were used for validation experiments. 22Rv1 and C4-2 are established prostate cancer models with AR-pathway relevance and CRPC-associated features (Sramkoski et al., 1999; Wu et al., 1994). 22Rv1 and RWPE-1 cells were obtained from ATCC, and LNCaP C4-2 cells were obtained from Cytion. 22Rv1 and C4-2 cells were maintained in RPMI-1640 medium supplemented with 10% fetal bovine serum and 1% penicillin-streptomycin. RWPE-1 cells were cultured in Keratinocyte Serum-Free Medium supplemented according to the supplier’s instructions. Cells were incubated at 37 °C in 5% CO2, used during logarithmic growth, and routinely tested for *mycoplasma* contamination.

### 2.12 Establishment of SLPI-overexpressing stable 22Rv1 and LNCaP C4-2 cell lines

To establish SLPI-overexpressing stable cell lines, the full-length human SLPI coding sequence was cloned into a lentiviral expression vector carrying a puromycin-resistance cassette; the corresponding empty vector was used as the negative control. Lentiviral particles were generated in HEK293T cells by co-transfecting the SLPI overexpression or control vector with psPAX2 and pMD2.G using Lipofectamine 3000. Viral supernatants were collected at 48 h and 72 h, filtered through a 0.45 μm membrane, and used to infect 22Rv1 and C4-2 cells in the presence of Polybrene. After puromycin selection, stable cell populations were expanded, and SLPI overexpression efficiency was confirmed by qRT-PCR before functional assays.

### 2.13 Quantitative real-time PCR

Total RNA was extracted using the RNeasy Mini Kit according to the manufacturer’s instructions. RNA concentration and purity were assessed by spectrophotometry, and qualified RNA was reverse-transcribed using the PrimeScript RT reagent Kit with gDNA Eraser. qRT-PCR was performed using TB Green Premix Ex Taq II on a real-time PCR system. Each 20 μL reaction contained 10 μL of 2x TB Green Premix, 0.4 μL of each primer, 2 μL of cDNA template, and nuclease-free water. Amplification was performed at 95 °C for 30 s, followed by 40 cycles of 95 °C for 5 s and 60 °C for 30 s. GAPDH was used as the internal reference gene, and relative expression was calculated using the 2ΔΔCt method (Livak and Schmittgen, 2001). Each experiment included at least three independent biological replicates.

GAPDH-F: GAC​AGT​CAG​CCG​CAT​CTT​CT

GAPDH-R: GCG​CCC​AAT​ACG​ACC​AAA​TC

SLPI-F: ATG​TGT​GGG​AAA​TCC​TGC​GT

SLPI-R: GGA​AAG​GAC​CTG​GAC​CAC​AC

KLK3-F: CCA​CAC​CCG​CTC​TAC​GAT​ATG​A

KLK3-R: CCC​AGA​ATC​ACC​CGA​GCA​G

TMPRSS2-F: ACT​GTA​AGG​TGC​TTG​CTC​CC

TMPRSS2-R: TTG​CCT​CAC​CTT​TGG​TCC​TC

FKBP5-F: CGG​CGA​CAG​GTT​CTC​TAC​TT

FKBP5-R: CGC​CTG​CAT​GTA​TTT​GCC​TC

NKX3.1-F: CCGGGCGGGTGCATT

NKX3.1-R: AAC​TCG​ATC​ACC​TGA​GTG​TGG

### 2.14 Colony formation assay

For colony formation assays, 22Rv1 and C4-2 cells were seeded into 6-well plates at 500–1,000 cells per well. After attachment, cells were treated with 5 μM enzalutamide or vehicle where indicated, and medium was refreshed every 3 days. After 10–14 days, colonies were fixed with 4% paraformaldehyde and stained with 0.1% crystal violet. Colonies containing more than 50 cells were photographed and counted. Colony formation was expressed relative to the corresponding control group, and all experiments were performed with at least three independent biological replicates.

### 2.15 Cell proliferation assay by CCK-8

Cell proliferation was measured using Cell Counting Kit-8 in 96-well plates. Vector-control and SLPI-overexpressing 22Rv1 and C4-2 cells were seeded at 2 × 10^3^ to 3 × 10^3^ cells per well in 100 μL of complete medium. At 0, 24, 48, 72, and 96 h, 10 μL of CCK-8 reagent was added, and absorbance at 450 nm was measured after incubation. Cell viability was expressed as the optical density value at each time point. Each condition included technical replicates, and experiments were independently repeated at least three times.

### 2.16 CCK-8 assay for enzalutamide sensitivity

To assess enzalutamide sensitivity, vector-control and SLPI-overexpressing 22Rv1 and C4-2 cells were seeded into 96-well plates and allowed to attach overnight. Cells were treated with 0, 1, 5, 10, or 20 μM enzalutamide for the indicated period. After drug exposure, CCK-8 reagent was added, absorbance at 450 nm was measured, and cell viability was calculated relative to the untreated control group. Each assay was performed with replicate wells and repeated independently at least three times.

### 2.17 Wound healing assay

Cell migration was assessed by wound healing assay. Vector-control and SLPI-overexpressing 22Rv1 and C4-2 cells were seeded into 6-well plates and cultured to approximately 90%–100% confluence. A linear wound was generated with a sterile 200 μL pipette tip. Detached cells were removed with PBS, and cultures were maintained in serum-free medium to minimize proliferation-associated wound closure. Images were captured at 0 h and 48 h, wound width was measured using ImageJ, and migration was calculated as the percentage of wound closure relative to the initial wound area. Experiments were independently repeated at least three times.

### 2.18 siRNA transfection

Transient siRNA transfection was performed using Lipofectamine RNAiMAX according to the manufacturer’s instructions. 22Rv1 and C4-2 cells were seeded into 6-well plates and cultured to approximately 30%–50% confluence. AR-targeting siRNA or negative-control siRNA was diluted in Opti-MEM, mixed with RNAiMAX, and added to the cells at a final siRNA concentration of 50 nM. After 4–6 h, the medium was replaced with fresh complete medium, and cells were collected after 24–48 h for qRT-PCR. AR knockdown efficiency was verified before downstream analyses.

### 2.19 Annexin V/PI apoptosis assay

Apoptosis was measured using Annexin V-FITC/PI staining according to the manufacturer’s instructions, a flow-cytometric approach based on phosphatidylserine exposure and membrane integrity (Vermes et al., 1995). Vector-control and SLPI-overexpressing 22Rv1 and C4-2 cells were treated with 10 μM enzalutamide for 48 h where indicated. Floating and adherent cells were collected, washed with cold PBS, resuspended in binding buffer, and stained with Annexin V-FITC and propidium iodide for 15 min at room temperature in the dark. Samples were analyzed by flow cytometry, and apoptotic cell proportions were quantified from Annexin V/PI staining. Experiments were independently repeated at least three times.

### 2.20 Induction of AR signaling by CSS deprivation followed by DHT supplementation and subsequent AR knockdown

To assess AR-dependent regulation of SLPI, an androgen-deprivation/re-stimulation model was established in 22Rv1 and C4-2 cells. Cells were cultured in medium containing 10% charcoal-stripped serum (CSS) for 48 h to reduce steroid-hormone signaling, followed by stimulation with 10 nM dihydrotestosterone (DHT) for 24 h to reactivate AR signaling. Total RNA was collected for qRT-PCR analysis of canonical AR target genes, including KLK3, TMPRSS2, FKBP5, and NKX3.1, together with SLPI.

To further validate AR-dependent regulation, cells were transfected with AR-targeting siRNA or negative-control siRNA using Lipofectamine RNAiMAX. Where indicated, cells were subjected to charcoal-stripped serum pretreatment followed by DHT supplementation before siRNA-mediated AR knockdown. After 24–48 h, RNA was collected for qRT-PCR analysis of AR and SLPI expression. Relative expression values were normalized to GAPDH.

### 2.21 Statistical analysis

Statistical analyses were performed using R and GraphPad Prism. For bioinformatics analyses, two-group comparisons were evaluated using the Wilcoxon rank-sum test, and multiple-group comparisons were evaluated using the Kruskal–Wallis test unless otherwise specified. Correlations were assessed using Spearman analysis. Differential expression was analyzed using Seurat with Benjamini–Hochberg adjustment for multiple testing. Survival differences were evaluated by Kaplan-Meier analysis and log-rank testing, and Cox proportional hazards models were used to estimate hazard ratios. Experimental data are presented as mean ± SD from at least three independent experiments. Two-group experimental comparisons were analyzed using unpaired Student’s t-test, and multi-group comparisons were analyzed using one-way ANOVA as appropriate. Two-sided P values <0.05 were considered statistically significant.

## 3 Results

### 3.1 Single-cell transcriptomic landscape of localized prostate cancer and mCRPC

**FIGURE 1:**
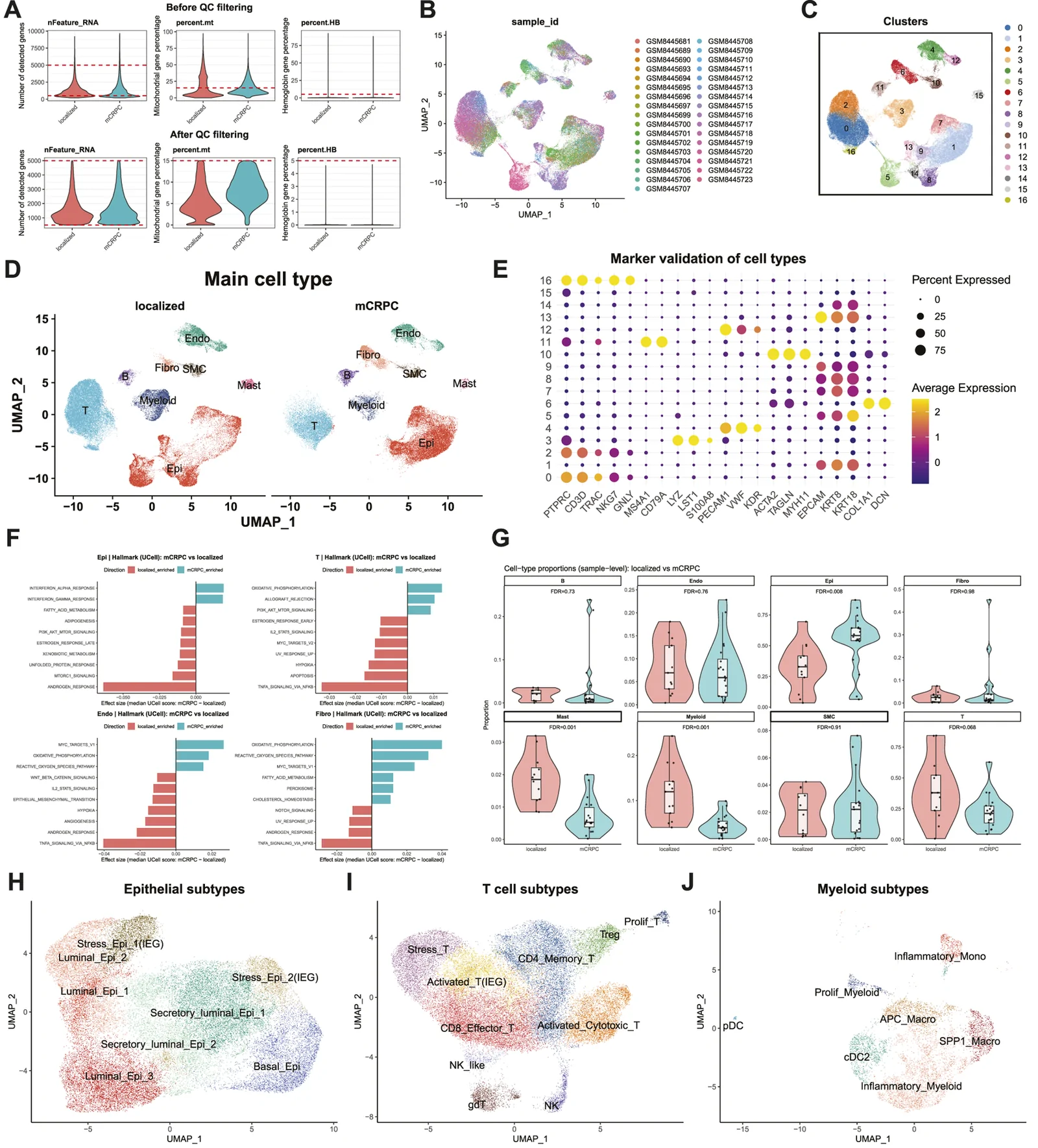
Single-cell transcriptomic landscape of localized prostate cancer and metastatic castration-resistant prostate cancer. **(A)** Quality control metrics before and after filtering, including number of detected genes, mitochondrial gene percentage, and hemoglobin gene percentage. Dashed lines indicate the filtering thresholds used for cell inclusion. **(B)** UMAP visualization colored by sample origin showing integration across samples. **(C)** UMAP plot showing unsupervised clustering of all cells. **(D)** Major cell type annotation in localized prostate cancer and mCRPC samples. **(E)** Dot plot showing canonical marker genes used for cell type annotation. Dot size indicates percentage of expressing cells and color intensity indicates average expression level. **(F)** Hallmark pathway activity differences between localized tumors and mCRPC across major cell types. Pathway names are from MSigDB Hallmark gene sets; underscore-formatted labels correspond to the standard Hallmark pathway names. **(G)** Comparison of cell type proportions between localized prostate cancer and mCRPC at the sample level. **(H)** UMAP visualization of epithelial cell subtypes. **(I)** UMAP visualization of T cell subtypes. **(J)** UMAP visualization of myeloid cell subtypes.

We first analyzed single-cell RNA-sequencing data from localized prostate cancer and mCRPC to define cellular changes associated with disease progression. After quality-control filtering, retained cells were used for downstream analysis (Figure 1A). UMAP visualization showed transcriptional heterogeneity across samples and effective integration (Figure 1B). Major compartments were annotated as epithelial cells, T cells, B cells, myeloid cells, endothelial cells, fibroblasts, smooth muscle cells, and mast cells (Figures 1C–E). Pathway analysis revealed coordinated changes across epithelial, immune, and stromal compartments (Figure 1F). Cell proportion analysis highlighted immune shifts, particularly in myeloid and mast-cell compartments (Figure 1G). Subclustering further resolved epithelial states, T-cell states, and heterogeneous myeloid populations (Figures 1H–J), providing a cell-resolved basis for subsequent analyses of therapeutic sensitivity and resistance.

### 3.2 Cell-cell communication remodeling during prostate cancer progression

**FIGURE 2:**
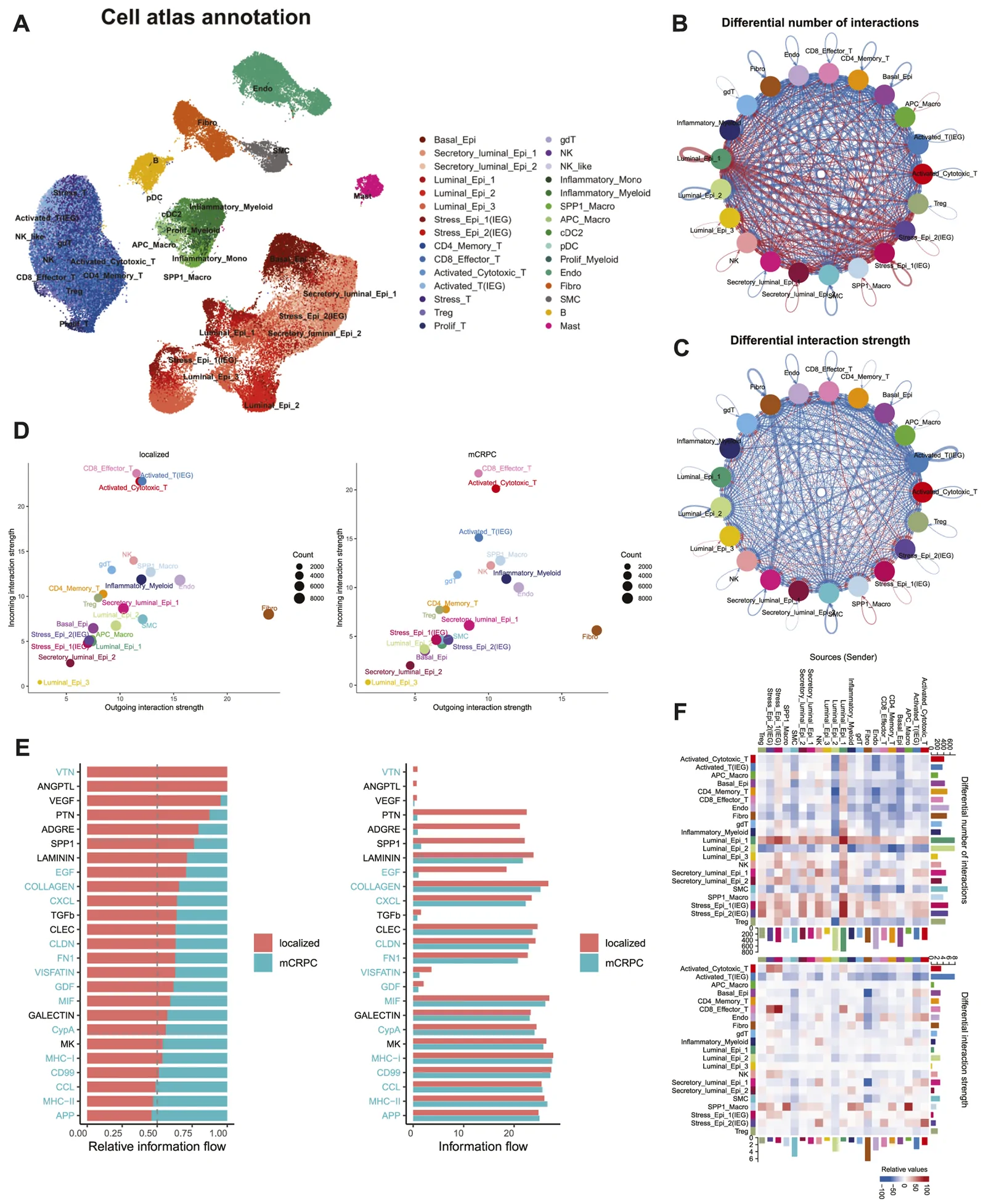
Inferred cell-cell communication landscape in localized prostate cancer and mCRPC. **(A)** Cell atlas showing annotated epithelial, immune, and stromal cell populations used for communication analysis. **(B)** Differential number of CellChat-inferred cell-cell interactions between localized prostate cancer and mCRPC. Red edges indicate increased interactions and blue edges indicate decreased interactions. **(C)** Differential interaction strength between cell populations. **(D)** Comparison of outgoing and incoming interaction strength across cell types in localized tumors and mCRPC. Dot size indicates interaction counts. **(E)** Relative information flow and overall information flow of signaling pathways comparing localized tumors and mCRPC. **(F)** Heatmaps showing differential interaction numbers and interaction strength across cell populations. These panels summarize transcriptome-inferred ligand-receptor programs and do not by themselves establish direct SLPI-dependent communication mechanisms.

We next examined intercellular communication to determine how disease progression reshapes inferred signaling between epithelial cells and non-malignant ecosystem compartments. CellChat analysis identified dense ligand-receptor networks in both localized prostate cancer and mCRPC (Figure 2A). Comparisons of interaction number and strength revealed broad reorganization of cellular crosstalk (Figures 2B,C). Epithelial and immune populations showed prominent outgoing signaling, whereas myeloid and stromal compartments displayed strong incoming signaling patterns (Figure 2D). Differential information-flow analysis identified changes in extracellular matrix organization, growth-factor signaling, immune regulation, and inflammatory pathways (Figures 2E,F). Together, these analyses suggest that CRPC progression is accompanied by broad signaling rewiring, while the inferred communication networks still require direct experimental validation.

### 3.3 Epithelial cell state remodeling during prostate cancer progression

**FIGURE 3:**
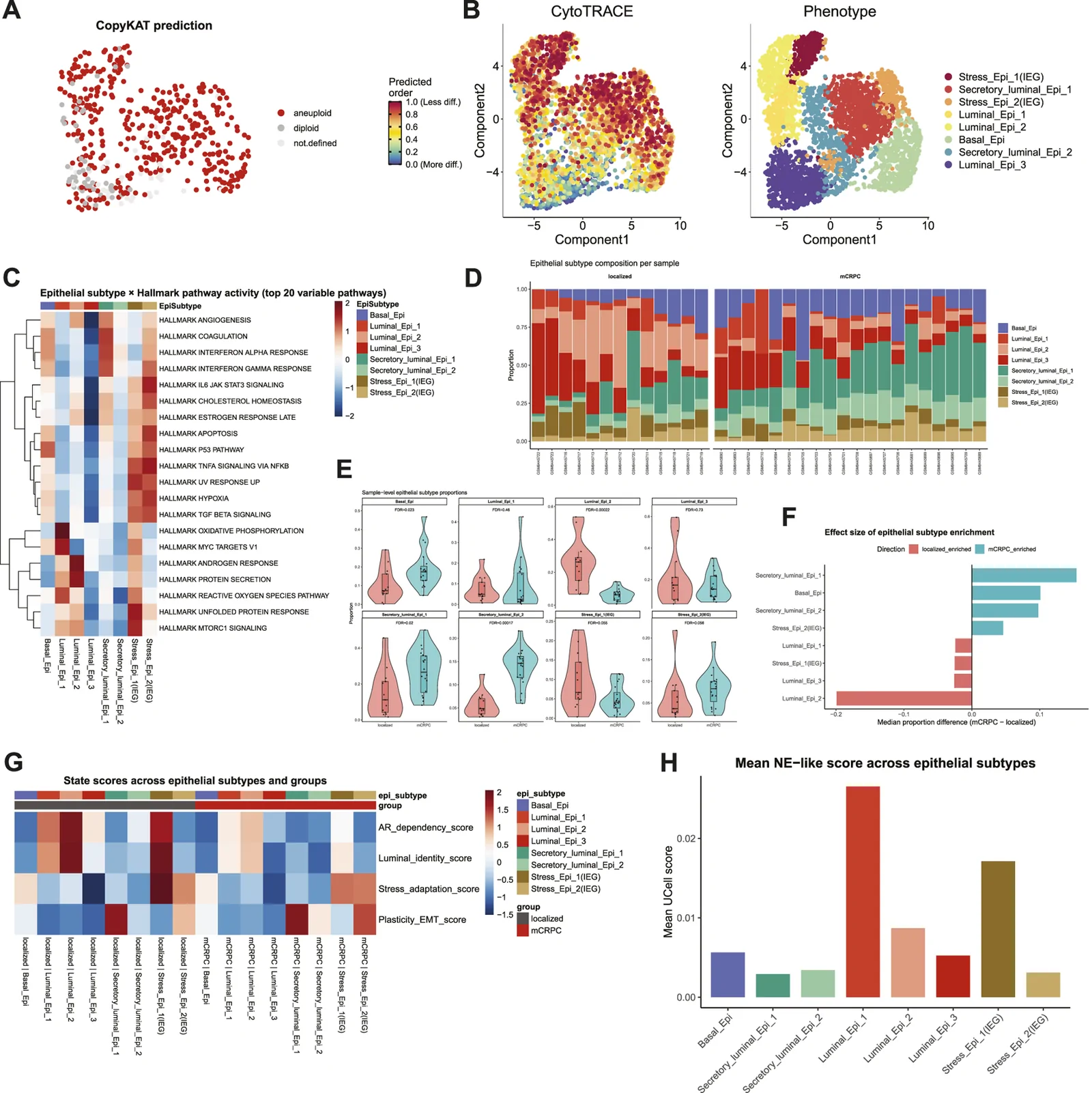
Functional heterogeneity and state remodeling of epithelial cells during prostate cancer progression. **(A)** CopyKAT prediction showing inferred copy number variation states of epithelial cells. Aneuploid cells indicate putative malignant cells, whereas diploid cells are considered likely non-malignant. **(B)** CytoTRACE analysis showing differentiation potential across epithelial cells and distribution across epithelial phenotypes. **(C)** Heatmap showing Hallmark pathway activity across epithelial subtypes. **(D)** Stacked bar plots showing epithelial subtype composition across samples in localized prostate cancer and mCRPC. **(E)** Comparison of epithelial subtype proportions between localized tumors and mCRPC. **(F)** Effect size of epithelial subtype enrichment between localized tumors and mCRPC. **(G)** Heatmap showing functional state scores including AR dependency, luminal identity, stress adaptation, and EMT plasticity across epithelial subtypes. **(H)** Mean neuroendocrine-like score across epithelial subtypes. Scores were calculated using UCell-based rank scoring of predefined gene programs.

Because malignant epithelial cells are the direct targets of AR-directed pharmacology, we focused on epithelial remodeling. CopyKAT analysis supported the putative malignant identity of aneuploid epithelial cells (Figure 3A), and CytoTRACE analysis showed heterogeneity in differentiation potential (Figure 3B). Hallmark scoring revealed that stress-associated epithelial states were enriched for inflammatory response, hypoxia, and apoptosis-related programs, whereas luminal states showed stronger androgen-response and metabolic activity (Figure 3C). Compared with localized tumors, mCRPC samples showed reduced luminal composition and increased stress-associated epithelial states (Figures 3D–F). Functional scoring showed lower AR dependency and luminal identity together with higher stress-adaptation and EMT/plasticity scores in stress-associated states (Figures 3G,H). These results indicate that epithelial adaptation is a prominent feature of the mCRPC state.

### 3.4 Pseudotime trajectory analysis suggests potential epithelial state transitions during prostate cancer progression

**FIGURE 4:**
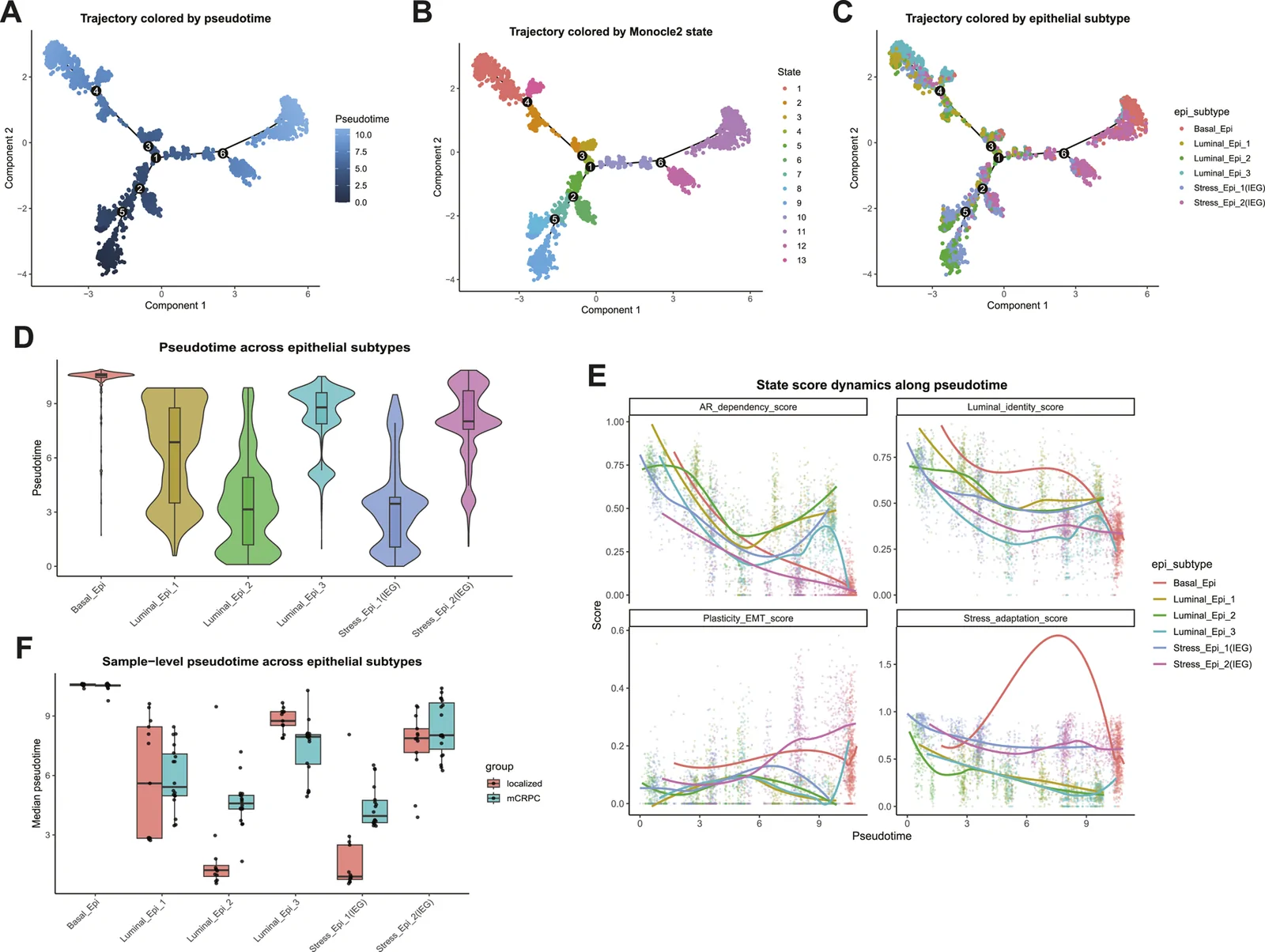
Pseudotime trajectory analysis of potential epithelial cell state transitions. **(A)** Pseudotime trajectory of epithelial cells inferred using Monocle2. Cells are colored according to pseudotime values. **(B)** Trajectory colored by Monocle2 cell states. **(C)** Distribution of epithelial subtypes along the trajectory. **(D)** Comparison of pseudotime distributions across epithelial subtypes. **(E)** Dynamic changes of functional state scores including AR dependency, luminal identity, EMT plasticity, and stress adaptation along pseudotime. **(F)** Sample-level comparison of pseudotime distributions across epithelial subtypes between localized prostate cancer and mCRPC. Pseudotime analysis provides a computational ordering of transcriptional states and should not be interpreted as direct lineage tracing.

Trajectory analysis further suggested potential epithelial state transitions during progression. Monocle2 reconstruction revealed a branched structure with multiple inferred routes (Figures 4A,B). Luminal epithelial populations occupied intermediate trajectory regions, whereas stress-associated states were enriched toward terminal branches (Figures 4C,D). Across pseudotime, AR dependency and luminal identity decreased along portions of the trajectory, while stress adaptation and EMT/plasticity increased (Figure 4E). Disease-group comparisons showed shifts in pseudotime distributions between localized prostate cancer and mCRPC (Figure 4F). These findings are consistent with a shift toward epithelial states that are more stress-tolerant and less dependent on canonical AR-luminal identity, although pseudotime analysis does not prove a direct lineage transition.

### 3.5 Identification of candidate genes associated with epithelial state remodeling

**FIGURE 5:**
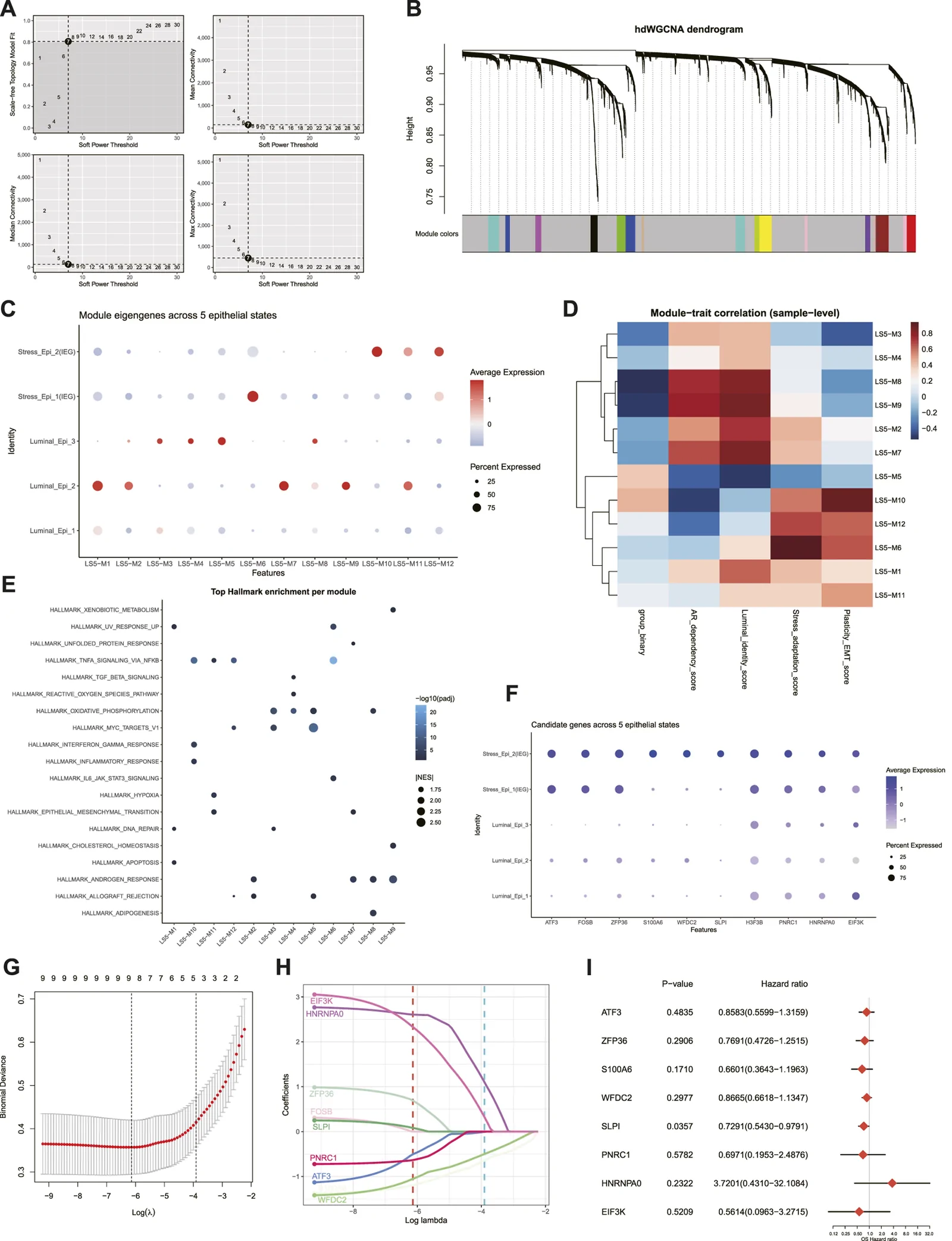
Identification of candidate genes associated with epithelial state remodeling. **(A)** Determination of the soft-thresholding power for hdWGCNA network construction. **(B)** hdWGCNA dendrogram showing gene modules identified based on co-expression patterns. **(C)** Module eigengene expression across epithelial subtypes. **(D)** Correlation heatmap showing associations between gene modules and epithelial functional traits. **(E)** Hallmark pathway enrichment analysis of module genes. **(F)** Expression patterns of candidate genes across epithelial subtypes. **(G)** LASSO Cox regression cross-validation curve for selecting the optimal lambda value. **(H)** LASSO coefficient profiles of candidate genes. **(I)** Forest plot showing associations between candidate gene expression and survival outcomes. SLPI was prioritized because it combined epithelial/immunoregulatory biology, candidate-gene status, and the clearest nominal outcome association among the tested genes.

To identify regulators of these adaptive states, we performed hdWGCNA on epithelial cells. Co-expression modules were constructed after soft-threshold selection (Figures 5A,B), and module eigengenes revealed subtype-specific programs (Figure 5C). Module-trait correlation analysis linked selected modules to AR dependency, luminal identity, stress adaptation, and EMT/plasticity (Figure 5D). Enrichment analysis showed that these modules involved stress response, inflammatory signaling, oxidative phosphorylation, and epithelial-mesenchymal transition (Figure 5E). Intersecting module-associated genes with epithelial expression patterns identified candidates including ATF3, FOSB, ZFP36, S100A6, WFDC2, SLPI, PNRC1, HNRNPA0, and EIF3K (Figure 5F). Candidate genes were further evaluated using LASSO Cox regression, including cross-validation for optimal lambda selection and coefficient profiling (Figures 5G,H). Among these candidates, SLPI was selected for functional testing because it is a secreted epithelial/immunoregulatory factor, has reported relevance to prostate cancer biology, and showed a nominally significant protective association in the TCGA-PRAD survival analysis shown in Figure 5I.

### 3.6 SLPI suppresses proliferation and migration of prostate cancer cells

**FIGURE 6:**
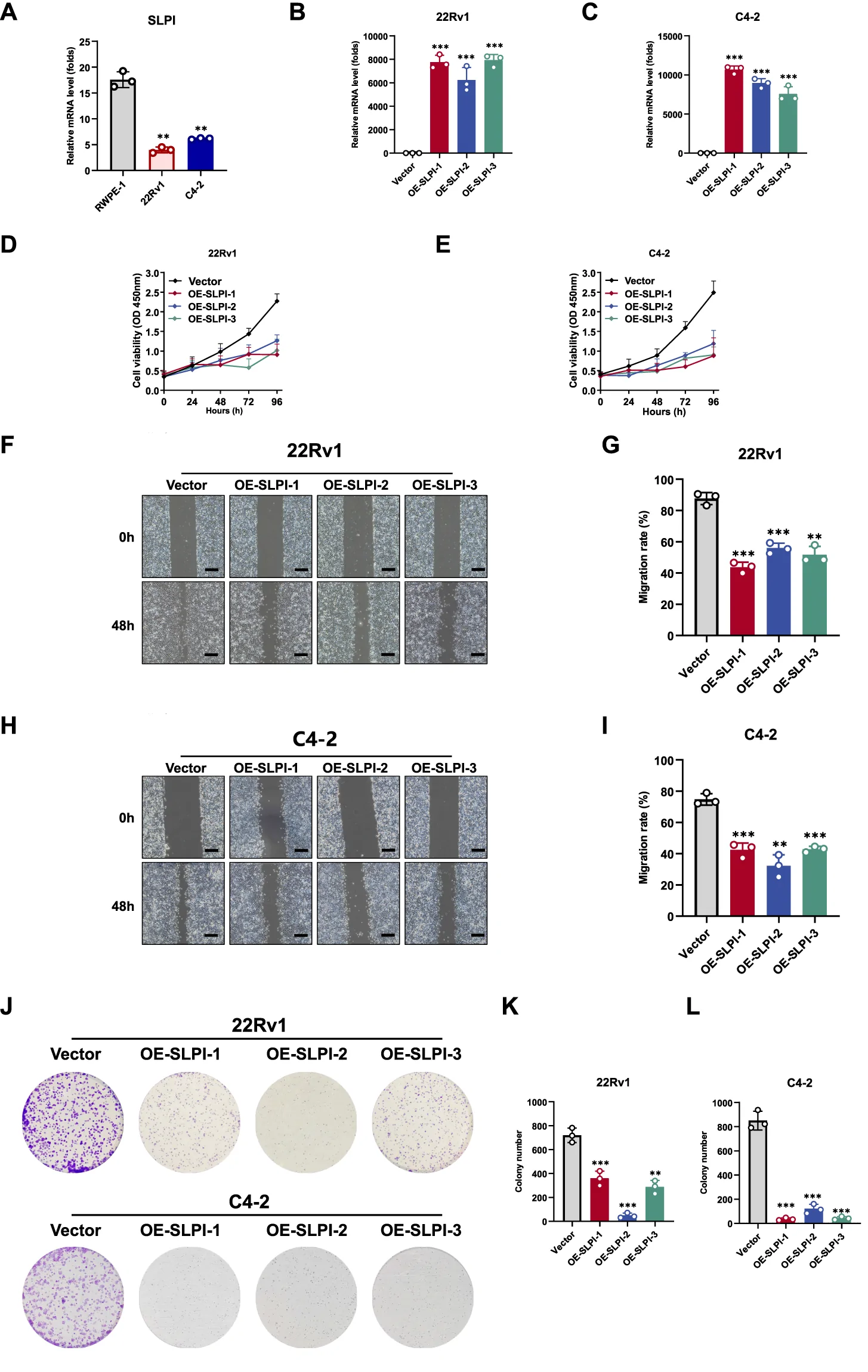
SLPI suppresses proliferation and migration of prostate cancer cells. **(A)** qPCR analysis showing SLPI expression in normal prostate epithelial cells (RWPE-1) and prostate cancer cell lines (22Rv1 and C4-2). **(B,C)** qPCR validation of SLPI overexpression efficiency in 22Rv1 **(B)** and C4-2 **(C)** cells. **(D,E)** CCK-8 assays showing reduced proliferation of SLPI-overexpressing 22Rv1 **(D)** and C4-2 **(E)** cells. **(F,G)** Representative wound healing images and quantification showing reduced migration of SLPI-overexpressing 22Rv1 cells. **(H,I)** Representative wound healing images and quantification showing reduced migration of SLPI-overexpressing C4-2 cells. **(J,K)** Representative colony formation images and quantification for 22Rv1 cells. **(L)** Colony formation quantification for C4-2 cells. Data are presented as mean ± SD from three independent experiments. *P < 0.05, **P < 0.01, ***P < 0.001.

We next examined SLPI experimentally because it connected the single-cell analysis, network prioritization, published prostate cancer biology, and clinical association. qRT-PCR showed lower SLPI expression in 22Rv1 and C4-2 prostate cancer cells than in RWPE-1 cells (Figure 6A). Stable overexpression was confirmed in both cancer cell lines (Figures 6B,C). SLPI overexpression reduced proliferation in CCK-8 assays (Figures 6D,E), inhibited wound closure in migration assays (Figures 6F–I), and suppressed colony formation (Figures 6J–L). These data indicate that SLPI restrains malignant phenotypes in the tested prostate cancer models.

### 3.7 AR signaling negatively regulates SLPI expression in prostate cancer cells

**FIGURE 7:**
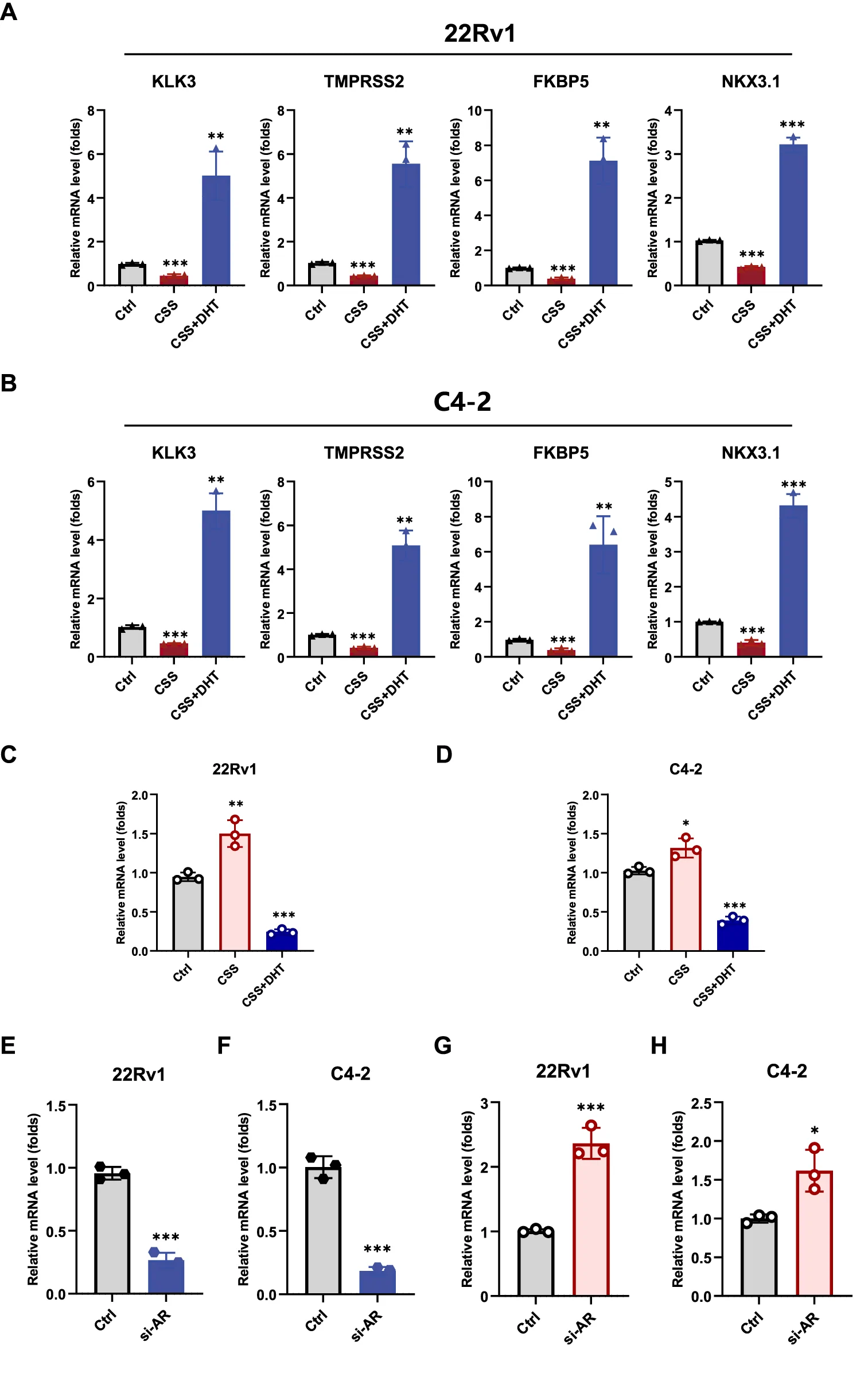
AR-pathway modulation is associated with reciprocal changes in SLPI expression in prostate cancer cells. **(A,B)** qPCR analysis showing expression of canonical AR target genes (KLK3, TMPRSS2, FKBP5, and NKX3.1) under control, CSS, and CSS + DHT conditions in 22Rv1 **(A)** and C4-2 **(B)** cells. **(C,D)** qPCR analysis showing SLPI expression under AR suppression (CSS) and AR reactivation (CSS + DHT) conditions in 22Rv1 **(C)** and C4-2 **(D)** cells. **(E,F)** qPCR validation showing reduced AR expression following siRNA-mediated knockdown in 22Rv1 **(E)** and C4-2 **(F)** cells. **(G,H)** qPCR analysis showing increased SLPI expression following AR knockdown in 22Rv1 **(G)** and C4-2 **(H)** cells. These experiments support AR-pathway-associated regulation of SLPI expression but do not establish direct AR binding. Data are presented as mean ± SD from three independent experiments. *P < 0.05, **P < 0.01, ***P < 0.001.

We then tested whether SLPI expression is linked to AR-pathway activity. Charcoal-stripped serum reduced canonical AR target genes, and DHT restored their expression, confirming effective AR modulation (Figures 7A,B). SLPI showed the reciprocal pattern: androgen deprivation increased SLPI expression, whereas DHT-mediated AR reactivation reduced SLPI expression (Figures 7C,D). AR knockdown further increased SLPI expression in both 22Rv1 and C4-2 cells (Figures 7E–H). These data indicate that SLPI is responsive to changes in androgen signaling, although they do not establish direct AR binding at the SLPI locus.

### 3.8 SLPI enhances sensitivity to enzalutamide in prostate cancer cells

**FIGURE 8:**
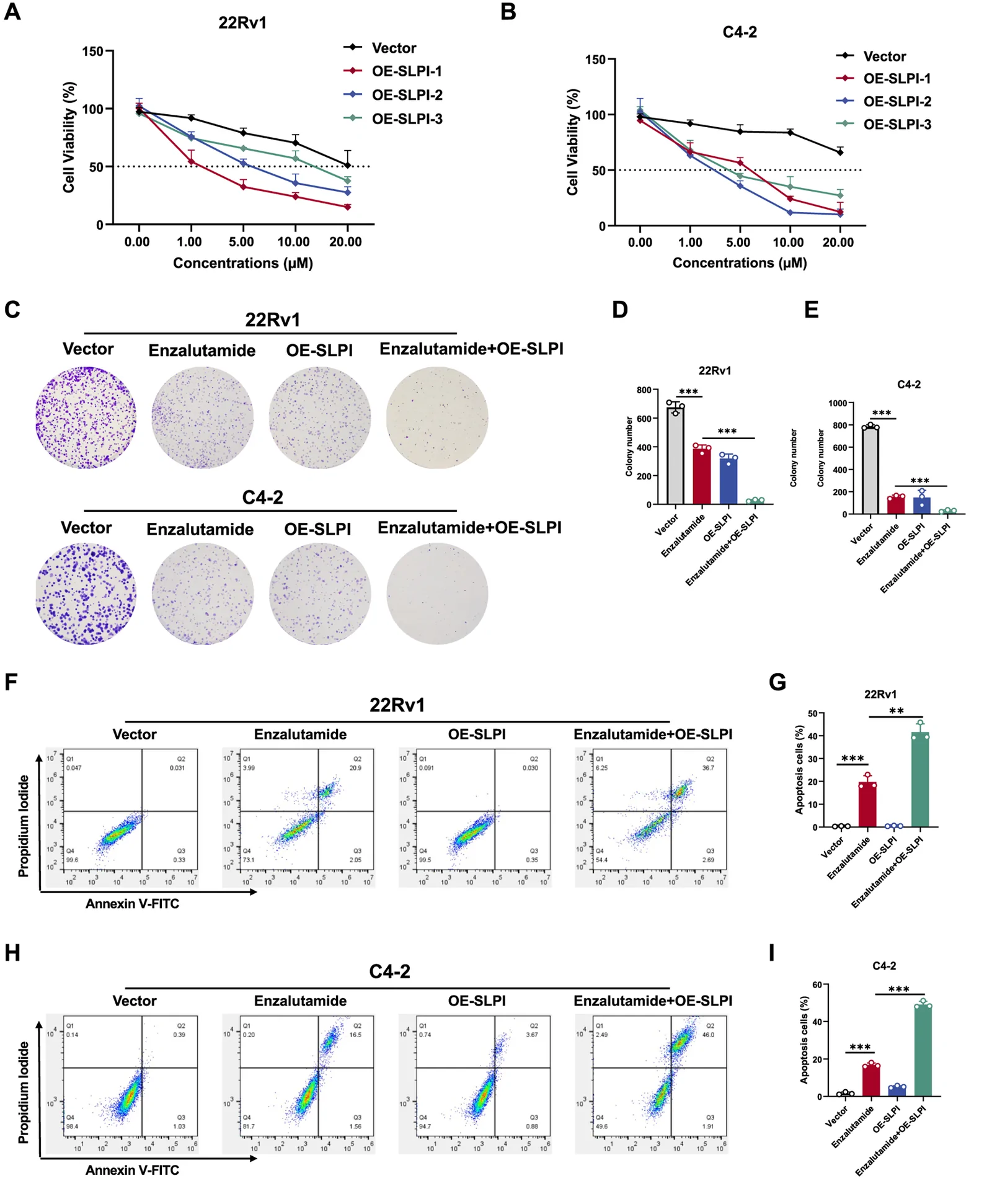
SLPI overexpression enhances sensitivity of prostate cancer cells to enzalutamide *in vitro*. **(A,B)** CCK-8 assays showing that SLPI overexpression enhances the inhibitory effect of enzalutamide on cell viability in 22Rv1 **(A)** and C4-2 **(B)** cells. **(C)** Representative colony formation images of 22Rv1 and C4-2 cells treated with vector, enzalutamide, OE-SLPI, or enzalutamide + OE-SLPI. **(D,E)** Quantification of colony numbers in 22Rv1 **(D)** and C4-2 **(E)** cells. **(F,H)** Representative Annexin V-FITC/PI flow cytometry plots showing apoptosis in 22Rv1 **(F)** and C4-2 **(H)** cells. **(G,I)** Quantification of apoptotic cell proportions in 22Rv1 **(G)** and C4-2 **(I)** cells. Data are presented as mean ± SD from three independent experiments. *P < 0.05, **P < 0.01, ***P < 0.001.

Finally, we evaluated whether SLPI affects pharmacologic response to enzalutamide. Across increasing drug concentrations, SLPI-overexpressing cells showed greater loss of viability than vector-control cells (Figures 8A,B). In colony formation assays, SLPI overexpression and enzalutamide together produced stronger suppression of clonogenic growth than either intervention alone (Figures 8C–E). Annexin V/PI analysis showed that SLPI overexpression increased apoptosis and further enhanced enzalutamide-associated apoptotic cell death (Figures 8F–I). These results suggest that SLPI can modulate enzalutamide response *in vitro*.

## 4 Discussion

This study identifies SLPI as an AR-pathway-associated, immunoregulatory epithelial factor related to cellular changes in CRPC and to enzalutamide sensitivity *in vitro*. The central question was pharmacologic as well as biological: how cell-type-specific states and intercellular communication may shape therapeutic sensitivity and resistance. By combining single-cell analysis with drug-response assays, we found that SLPI is associated with epithelial adaptation and treatment vulnerability, while recognizing that the present work does not define every causal step.

The single-cell data provide the biological context for this observation. mCRPC was characterized by altered epithelial-immune/stromal communication, immune and myeloid compartment changes, and increased stress-associated epithelial states (Chi et al., 2025). The functional assays focused on AR-directed treatment, whereas the single-cell analysis showed that epithelial drug response occurs in parallel with immune and stromal changes. The communication analysis remains transcriptomic and inferential; therefore, it should be interpreted as a source of mechanistic hypotheses, not as direct evidence that SLPI itself mediates cell-cell communication.

SLPI is relevant in this setting because it is both epithelial and immunoregulatory. Its roles in protease inhibition, antimicrobial defense, epithelial protection, and inflammatory control provide plausible routes through which it may affect tumor-cell stress tolerance and microenvironmental communication. Recent prostate cancer literature has emphasized the context-dependent nature of SLPI biology, including associations with prostate cancer progression and CRPC cell survival (Rosini et al., 2026; Zheng et al., 2016). In our experiments, SLPI overexpression suppressed proliferation, migration, and clonogenic growth, supporting a tumor-restraining role in the tested prostate cancer models. These observations suggest that reduced SLPI expression may accompany or enable epithelial adaptation during progression, although this possibility will require loss-of-function and protein-level validation.

The reciprocal relationship between AR signaling and SLPI provides a mechanistic bridge to CRPC pharmacology. Androgen deprivation and AR knockdown increased SLPI expression, whereas DHT-mediated AR reactivation suppressed SLPI. This pattern indicates that SLPI expression tracks AR-pathway state and may participate in the transcriptional remodeling that occurs when prostate cancer cells adapt to therapy. Because AR pathway inhibitors remain central to CRPC treatment, regulators that alter vulnerability to AR inhibition have direct pharmacologic relevance. Nevertheless, the current data do not determine whether AR controls SLPI directly through promoter/enhancer binding or indirectly through downstream transcriptional programs.

Functionally, SLPI overexpression enhanced enzalutamide-induced growth inhibition, colony suppression, and apoptosis. We therefore interpret SLPI as a candidate modulator of drug response rather than as a validated sensitizing factor. Within the broader resistance landscape, SLPI should be positioned alongside established mechanisms such as persistent AR signaling, AR-indifferent plasticity, lineage switching, stress-response programs, and microenvironmental immune remodeling rather than as a single dominant driver (Bluemn et al., 2017; Aggarwal et al., 2018; Labrecque et al., 2019; Fayi and Dera, 2025). The current evidence supports a role in enzalutamide sensitivity *in vitro* and justifies further work to determine whether SLPI itself, upstream AR control of SLPI, or downstream SLPI-associated pathways contribute to therapy-responsive epithelial states (Jiang C. et al., 2024).

Several limitations should be considered. Functional validation was performed in cell-line models using SLPI overexpression, and complementary loss-of-function experiments will be needed to determine whether endogenous SLPI is required for the observed phenotypes. The present study also did not assess SLPI protein abundance, invasive potential, *in vivo* tumor behavior, or patient-level enzalutamide response. Finally, the experiments do not define whether SLPI acts through direct AR-pathway regulation or through downstream stress-response, apoptotic, secretory, or microenvironment-related pathways (Wang et al., 2024). These limitations define the next steps while leaving the main conclusion intact: SLPI is a biologically plausible regulator of pharmacologic adaptation in CRPC models.

Overall, this study places SLPI within AR-regulated epithelial adaptation, immune-context changes, and enzalutamide sensitivity. The findings show how single-cell communication analysis and functional drug-response assays can be combined to study therapeutic sensitivity and resistance in prostate cancer.

## 5 Conclusion

In conclusion, SLPI is an AR-pathway-associated epithelial and immunoregulatory factor related to CRPC cellular adaptation and enzalutamide response. Single-cell analysis showed that progression to mCRPC involves altered epithelial-immune/stromal communication, immune/myeloid remodeling, reduced AR-luminal identity, and increased stress-associated epithelial programs. Functional validation demonstrated that SLPI overexpression suppresses malignant phenotypes and enhances enzalutamide-induced growth inhibition and apoptosis in prostate cancer cell-line models. These data nominate SLPI as a therapy-response-associated epithelial factor in prostate cancer and support further mechanistic validation.

## Data Availability

The original contributions presented in the study are included in the article/supplementary material, further inquiries can be directed to the corresponding author.
